# Aptamer Technologies in Neuroscience, Neuro-Diagnostics and Neuro-Medicine Development

**DOI:** 10.3390/molecules29051124

**Published:** 2024-03-02

**Authors:** Bang Wang, Firas Kobeissy, Mojtaba Golpich, Guangzheng Cai, Xiaowei Li, Reem Abedi, William Haskins, Weihong Tan, Steven A. Benner, Kevin K. W. Wang

**Affiliations:** 1 Department of Chemistry, University of Florida, Gainesville, FL 32611, USA; wangbang1213@163.com (B.W.); cpulixiaowei@gmail.com (X.L.); 2 The Foundation for Applied Molecular Evolution, 1501 NW 68th Terrace, Gainesville, FL 32605, USA; 3 Center for Neurotrauma, MultiOmics and Biomarkers (CNMB), Department of Neurobiology, Neuroscience Institute, Morehouse School of Medicine, Atlanta, GA 30310, USA; firasko@gmail.com (F.K.); mgolpich@msm.edu (M.G.); gcai@msm.edu (G.C.); 4 Department of Emergency Medicine, University of Florida, Gainesville, FL 32611, USA; 5 Brain Rehabilitation Research Center, Malcom Randall VA Medical Center, North Florida/South Georgia Veterans Health System, 1601 SW Archer Road, Gainesville, FL 32608, USA; 6 Center for Visual and Neurocognitive Rehabilitation (CVNR), Atlanta VA Health Care System, 1670 Clairmont Rd, Decatur, GA 30033, USA; 7 Department of Biochemistry and Molecular Genetics, American University of Beirut, Beirut 1107-2020, Lebanon; reemabedi@live.com; 8 Gryphon Bio, Inc., 611 Gateway Blvd. Suite 120 #253, South San Francisco, CA 94080, USA; will@gryphonbio.com; 9 Zhejiang Cancer Hospital, Hangzhou Institute of Medicine (HIM), The Chinese Academy of Sciences, Hangzhou 310022, China; tan@hnu.edu.cn

**Keywords:** aptamers, SELEX technology, therapeutic approaches, neuro-diagnostics development, neuro-medicine, neuroscience

## Abstract

Aptamers developed using in vitro Systematic Evolution of Ligands by Exponential Enrichment (SELEX) technology are single-stranded nucleic acids 10–100 nucleotides in length. Their targets, often with specificity and high affinity, range from ions and small molecules to proteins and other biological molecules as well as larger systems, including cells, tissues, and animals. Aptamers often rival conventional antibodies with improved performance, due to aptamers’ unique biophysical and biochemical properties, including small size, synthetic accessibility, facile modification, low production cost, and low immunogenicity. Therefore, there is sustained interest in engineering and adapting aptamers for many applications, including diagnostics and therapeutics. Recently, aptamers have shown promise as early diagnostic biomarkers and in precision medicine for neurodegenerative and neurological diseases. Here, we critically review neuro-targeting aptamers and their potential applications in neuroscience research, neuro-diagnostics, and neuro-medicine. We also discuss challenges that must be overcome, including delivery across the blood–brain barrier, increased affinity, and improved in vivo stability and in vivo pharmacokinetic properties.

## 1. Introduction

Aptamers are single-stranded (ss) oligonucleotides (RNA or DNA) that bind to their respective targets with high selectivity and affinity due to their unique three-dimensional structures. Generally, aptamers are chosen using Systematic Evolution of Ligands by Exponential Enrichment (SELEX), first used in 1990 [1,2]. Since then, fundamental advances have led to chemically engineered aptamers with higher binding affinities in more efficient and versatile workflows. Examples of advances include Slow Off-Rate Modified Aptamers (SOMAmer) [3,4], aptamers generated from expanded DNA “alphabets” [5,6], the adaptable amplification of synthetic oligonucleotides (evolution of sequence-defined aptamers) [7], and the high-throughput automation of the SELEX process [8].

Chemically engineered aptamers are envisioned as a family of potential artificial ligands for biomarker and drug discovery, diagnostics, drug delivery, and other therapeutic applications. Aptamers may also be used in process chemistry, for example, as chromatographic media for efficient and traceless purification of compounds with maximal biological integrity [9]. In the research laboratory, aptamers have been implemented to investigate binding phenomena in, for example, proteomics similar to what was envisioned in proteomics-based biomarkers [10,11,12,13,14]. Thus, aptamers as artificial ligands with a variety of unique properties are attractive for a spectrum of functions, from basic research to translational medicine.

Neurodegenerative disorders have been the second leading cause of death and the first leading cause of dementia in the past decade, which affect millions worldwide but lack effective treatment and prevention [15]. These diseases, including Alzheimer’s disease (AD), Parkinson’s disease (PD), Transmissible Spongiform Encephalopathies (TSE), Huntington’s Disease (HD), and prion protein diseases (PrD), are characterized by the accumulation of misfolded proteins in the CNS resulting from either genetic or environmental materials, as well as both factors.

Current research aims to manage neurodegenerative diseases by genetic intervention [16,17], as with siRNA drugs or gene therapy with, for example, adeno-associated virus (AAV) vectors. However, the safety and efficacy of gene therapies continue to involve challenging issues. Monoclonal antibody-based therapies are now available, such as treatment in multiple sclerosis (MS) [18] and the targeting of amyloid-beta (aducanumab [19]) for the treatment of AD. These applications have gained FDA approval, despite controversy, indicating that biological molecules can be developed to treat neurological and neurodegenerative disorders, and also reaffirming that it is feasible to treat neurodegenerative diseases by inhibiting protein post-translational modification (PTM), misfolding, and aggregation processes [20]. These approaches target a common pathological feature of these diseases: the accumulation of misfolded or PTM proteins and the resulting deposition of protein aggregates that appear to be toxic to nerve cells.

**Figure 1:**
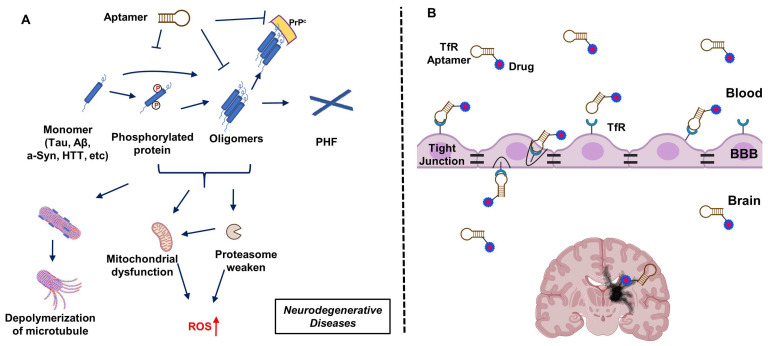
Aptamers applied in the treatment of neurodegenerative diseases. (**A**) Schematic illustration of aptamer mitigation of damage caused by the aggregation of neuro-related protein monomers (Tau, amyloid-β (Aβ), alpha-synuclein (a-Syn), Huntingtin (HTT)) that are often post-translationally modified or subject to proteolysis. (**B**) Schematic of the strategy of aptamer transfer across the blood–brain barrier (BBB) via transferrin receptor (TfR) mediated transcytosis. PHF: Poly(hexylene 2,5-furandicarboxylate); ROS: reactive oxygen species; PrPc: cellular prion protein.

In concept, aptamers, have a number of advantages over antibodies, including lower immunogenicity, smaller physical size, and better permeability [21]. Aptamers have evolved to bind neurologic disease-relevant proteins, inhibit neurodegenerative events resulting from PTM (e.g., phosphorylation), and/or prevent aggregation (Figure 1A). On the other hand, the transfer of biological molecules across the blood–brain barrier (BBB) remains the most challenging obstacle to treat brain diseases. On this front, exploiting the transferrin receptor (TfR) binding aptamer moiety provides a potentially viable path to the delivery of neuroactive aptamers or other drug molecules across the BBB (Figure 1B).

## 2. Discovery of Target-Specific Aptamers Using SELEX

**Figure 2:**
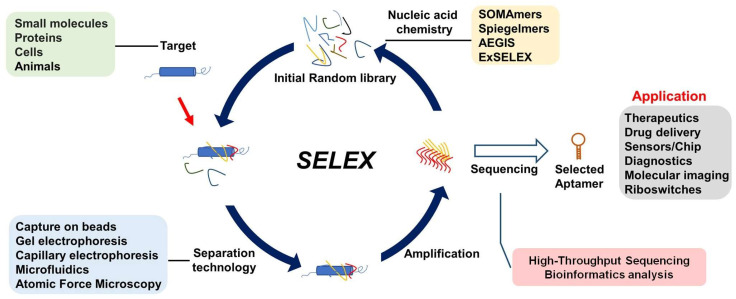
Schematic representation of SELEX (Systematic Evolution of Ligands by Exponential Enrichment).

Aptamers are traditionally generated using the Systematic Evolution of Ligands by Exponential Enrichment (SELEX), a laboratory analog of Darwinian evolution, applied to libraries of DNA and RNA nucleic acids to select functional oligonucleotides after iterative rounds of binding, purification, and amplification, as shown in Figure 2. Many research and development efforts have focused on standardization, streamlining, and increasing the flexibility of this process.

Briefly, an initial pool of random ligands with 10^14^~10^16^ variant sequences is incubated with a target of interest (bio-macromolecule, cell, or organic molecule, for example). The library is comprised of random sequences (typically containing 25–100 nucleotides) placed within primer regions. The library components that do not bind to the target are washed away. Then, components of the library that bind the target are eluted. These “survivors” are then amplified using PCR to construct a new pool, and the cycle is repeated. Mutation during PCR may create binders stronger than any in the original pool.

After confirming that the process has enriched the pools in binding sequences, often with a quantitative analysis of binding applied to the entire pool, sequences of surviving pool components are identified using a sequencing platform (e.g., using next-generation sequencing). The survivors are then ranked by their abundance in the pool. Top-ranked aptamers are then synthesized and studied as individual molecules.

To enhance the specificity of aptamers and eliminate off-target binding, several procedures can be employed during the SELEX process. One effective approach involves immobilizing potential off-targets, such as analogs or non-target molecules, onto solid supports like beads. These immobilized molecules are then used to pre-treat the aptamer pool, allowing non-specific binders to attach to these off-targets and be physically separated before the selection against the actual target. Another method involves using conjugate moieties that mimic the target structure to capture and remove aptamers with lower specificity. Additionally, employing counter-SELEX techniques, where aptamers binding to these negative targets are systematically removed, can further refine the specificity of the selected aptamers [22,23].

RNA-based SELEX procedures [24,25] require an additional step to convert RNA to its cDNA by reverse transcriptase followed by a PCR amplification step. Then, the cDNA is transcribed to RNA by T7 RNA polymerase to produce the pool for the next round. Apart from these critical steps, the other components of SELEX are flexible, and may be customized for more efficient selection. We summarize and highlight the significance of a variety of steps that empower SELEX for specific research applications in Figure 2.

As discussed above, to improve SELEX for the end-to-end bio-application of aptamer binding to target with high affinity and specificity, the SELEX principle has been adapted to the specific demands of each application, including but not limited to those shown in Figure 2. These applications are categorized below.

The first categorization of SELEX is based on the classification of targets, where different responses are observed for small molecules [26,27] (biological molecules, food, environmental toxins, fluorophores, and synthetic pharmaceuticals), proteins [28] (mammalian proteins, infectious agents, others), cells [29] (cancer cells, pathogenic microbes), whole viruses [30], and tissues or animals [31].

Different categories of SELEX are distinguished by different strategies for isolating potential aptamers from the library. The most common classical technique used in separation exploits solid phase capture. A His-tag or biotin-labeled protein or other target molecule is immobilized on the support via metal or streptavidin which, in turn, is supported on a magnetic bead or a non-magnetic matrix, such as agarose, Sephadex, or Sepharose. Library components that do not bind, or bind with low affinity under the washing conditions, are removed, while binders remain attached to the surface via a chain where aptamer binds to the target which is attached to a tag (biotin, Poly(histidine)) which binds to the support. Following this approach, hundreds of aptamers have been generated to bind various targets.

Other technologies have been applied in the SELEX separation step. These include capillary electrophoresis [32]; surface plasmon resonance (SPR) immobilization [33]; nitrocellulose filter binding [34]; gel electrophoresis [35]; micro free flow electrophoresis [36]; atomic force microscopy [37,38]; particle display SELEX [39]; microarray-based SELEX [40]; capture SELEX [41]; Non-SELEX [42]; High-fidelity SELEX (Hi-Fi) [43]; Microfluidic SELEX [44]; and IP-SELEX [45]. We recently developed a Functional SELEX-Based Selection of Tau Oligomerization-Inhibiting Aptamers [46].

**Figure 3:**
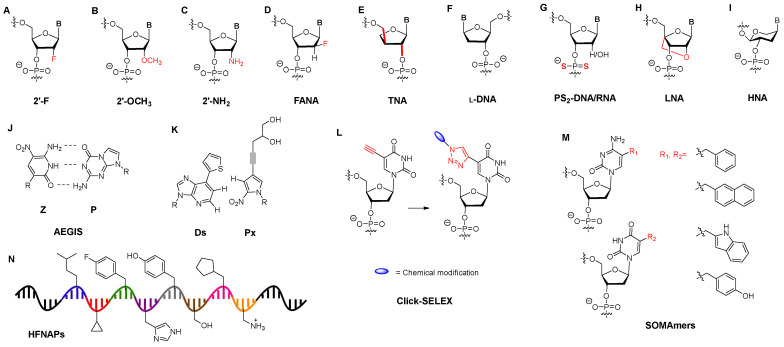
Chemical modifications of DNA or RNA building blocks for aptamers. (**A**) 2′-deoxy-2′-fluoro modification; (**B**) 2′-deoxy-2′-methoxy modification; (**C**) 2′-deoxy-2′-amino modification; (**D**) 2′-deoxy-2′-fluoroarabino nucleic acid; (**E**) TNA, α-L threose nucleic acid; (**F**) Spiegelmers (L-DNA); (**G**) phosphonodithioates (PS2); (**H**) locked nucleic acid (LNA); (**I**) 1,5-anhydrohexitol nucleic acid (HNA); (**J**) Artificially Expanded Genetic Information System-SELEX; (**K**) Genetic alphabet Expansion for SELEX (ExSELEX); (**L**) Click-SELEX; (**M**) Slow Off-rate Modified Aptamers (SOMAmers); (**N**) Highly functionalized nucleic acid polymers (HFNAPs).

SELEX strategies can also be categorized by the type of biopolymer presented in the library. DNA and RNA are often modified or replaced by recognizing that ssDNA and RNA can have poor biological stability, limited chemical diversity, and low binding affinity compared to antibodies. Hoping to rectify these limitations, chemical modifications of DNA or RNA building blocks (e.g., nucleotides, nucleosides, and nucleobases), as well as entirely unnatural building blocks, have been explored. The main alternatives to standard DNA and RNA include: 2′- deoxy-2′-fluoro RNA [47], 2′-O-methyl (or 2′-deoxy-2′-methoxy) RNA [48,49], 2′-deoxy-2′-NH2 RNA [50], 2′-deoxy-2′-fluoroarabino nucleic acid (FANA) [51], α-l-threose nucleic acid (TNA) [52], Spiegelmers (L-DNA) [53], phosphonodithioates (PS2) [54], locked nucleic acids (LNA) [55], 1,5-anhydrohexitol nucleic acid (HNA) [56], genetic alphabet expansion for SELEX (exSELEX) [57], Artificially Expanded Genetic Information System-SELEX (AEGIS-Selex) [6,58], Click-SELEX [59], Slow Off-rate Modified Aptamers (SOMAmers) [4], and highly functionalized nucleic acid polymers (HFNAPs) [7] (Figure 3).

Other technologies have also been incorporated into SELEX, such as bioinformatics analysis and high-throughput sequencing (HTS) technologies combined with SELEX (HT-SELEX [8,60]). These technologies facilitate the rapid identification of aptamers and help explore the landscape for molecular evolution. As an especially interesting case, Zhu and his group recently reported directly selected highly biostable mirror-image L-DNA aptamers with a mirror-image DNA polymerase [61]. This technology simplifies the Spiegelmers selection method, which requires a first step involving the mirror image of the target, which is difficult to obtain if the target is a large protein, and impossible if the target is a cell. Another interesting technology applies AI-driven machine learning for aptamer discovery [62]. This may provide a new avenue to develop high-quality aptamers (Box 1).

Box 1 **The direction of aptamer discovery.**

- Pacification or synthesis of targets suitable for aptamer selection, or efficient identification of the specific target when a mixture system is used in selection, as in Cell-Selex.
- Highly efficient separation of high binding affinity and specific aptamer from library pool; the ultimate goal is single-round selection.
- Chemically modified nucleic acid to improve limited chemical diversity, relatively low binding affinity, and resistance to nuclease. Development of the corresponding polymerase or methods for the amplification of modified nucleic acid.
- High-throughput selection of many targets at one time and high-throughput sequencing for aptamer evolution.
- AI-driven machine learning for aptamer discovery to avoid tedious manual operation and lower cost.
- Discovery of useful aptamers with more function not just binding to target; functionally directed selection offers a promising avenue to develop functional aptamers.

## 3. Comparing the Advantages and Disadvantages of Aptamers, Antibodies, and Small Molecules as Neuro-Therapeutics

Many drugs, such as penicillin and aspirin, have profoundly influenced the progress of human civilization. Since the birth of the pharmaceutical industry, chemically synthesized or naturally isolated small-molecule drugs have dominated much of the pharmacopeia. Such drug molecules have advantages, including scalable synthesis, rapid diffusion, facile translocation through plasma membranes, low-cost development and production, oral or parenteral administration, and broad applications such as enzymatic inhibitors or receptor agonists/antagonists in the cell.

Small-molecule drugs also have well-known limitations, many arising from the “hard slog” of medicinal chemistry. They are difficult to design de novo, in vitro assays may not mirror the biological environment where they act, and candidates that are successful in vitro may fail in vivo, because of bad pharmacodynamics, third-party interactions, and toxicity, for just a few of the reasons.

Consequently, attention has shifted to large molecule therapeutics, also known as biologics, in the 21st century. Representatives of biologics are monoclonal antibodies (mAbs), which bind to targets with high specificity and affinity (some may have dissociation constants, Kd’s, in the nM range). A monoclonal antibody (mAb) is a protein complex far larger than 20 amino acids, comprised of multiple chains that enable specific binding to target proteins or receptors. This structure allows for a diverse range of chemical interactions, facilitating precise targeting and potential catalytic activities. However, because mAbs are generally manufactured in fed-batch mode in large-scale bioreactors, the generation of consistent product quality at scale can be challenging, despite sophisticated upstream and downstream process development and analytics. Viral or bacterial contamination during the manufacturing process can also affect product quality. High immunogenicity and large size limit the bioavailability of mAbs, or entirely prevent their access to many important biological compartments, in particular for treating brain diseases, where they must also cross the BBB. All these shortcomings present significant barriers to the further development of mAbs for neurological and neurodegenerative disease, despite intense research and development interest [63,64].

Aptamers offer new therapeutic options that combine the advantages of small-molecule drugs and mAbs. For instance, aptamers are produced using a readily scalable chemical process with low cost. They are non-immunogenic, have a nanomolar affinity, and (often) exhibit high target specificity. Further, aptamers can be reversibly denatured and renatured, unlike protein mAbs, and the phosphodiester bond is chemically stable. Moreover, the conjugation of dyes or functional groups to aptamers is orthogonal and can be readily introduced during aptamer synthesis. Current aptamer designs also have several limitations, including the limited chemical functionality and diversity of natural genetic polymers (DNA and RNA). This provides few molecular “tools” to bind targets or to catalyze reactions. Unmodified aptamers often have poor stability in biological environments, being highly susceptible to plasma nuclease degradation. Further, aptamers are susceptible to renal filtration for clearance. Aptamers may also lose specific binding ability in a bioenvironment different from the environment where they were selected [65,66]. A comparison among small-molecule drugs, aptamers, and antibodies is shown in Table 1.

While aptamers offer several advantages, such as high specificity and low immunogenicity, there are potential side effects associated with their use. Although aptamer-related adverse events are rare in clinical evaluations to date, potential side effects have been identified, including nonspecific immune activation, where oligonucleotide-based therapies like aptamers might be mistakenly recognized by the immune system as pathogens, leading to unintended responses. Other toxicities may arise from polyanionic effects, unexpected tissue accumulation, and intensive chemical modification or conjugation, in particular with continuous or repeated administration of aptamer therapeutics [67,68,69]. Additionally, there is a risk of aptamers binding to unintended targets, causing off-target effects that vary in severity [69]. Hepatotoxicity is another concern, with certain chemical modifications to nucleic acids intended to enhance stability and potency having been shown to cause significant liver toxicity in animal models [70]. Allergic reactions, particularly to modifications such as polyethylene glycol (PEG) used to extend circulation time, have been documented, highlighting the need for screening for pre-existing antibodies to PEG [71]. These potential side effects underscore the importance of comprehensive preclinical and clinical testing for aptamer-based therapies. Thus, the benefits of aptamer therapy need to be weighed against these potential risks to ensure the optimal outcome for patients.

## 4. Aptamers Targeting Neuro-Medically Relevant Targets

Neuroprotein-targeting aptamers may also demonstrate outstanding potential for inhibiting protein aggregation associated with brain neuropathology, paralleling the success of aptamers in protein-binding applications. As discussed above, multiple aptamers have been selected against different CNS-relevant neuroproteins. This section will discuss some recent accomplishments of applying CNS-specific aptamers to the development of targeted neurotherapeutics. These crucial challenges have delayed the clinical translation of aptamers and the adoption of improvements in aptamer-based approaches in brain therapy.

Table 2 shows several DNA or RNA aptamers with good binding to CNS-specific or enriched protein targets, with affinities ranging from µM to nM. These include DNA and RNA aptamers that target β-Amyloid (Aβ) protein, Aβ42 dimer, Aβ40 oligomers, Tau protein for AD, and potentially AD-related disorders such as 4R tauopathies [72]. Further, α-synuclein protein and Prion protein (PrP) aptamers have been developed for PD and prion protein diseases [73]. In other studies, Toll-like receptor 4 (TLR4) and the regulator of calcineurin 1 (RCAN1) have been targeted for aptamer development as a potential treatment for Stroke disease [74] such as Acute ischemic stroke (AIS) [75,76]. For Amyotrophic Lateral Sclerosis (ALS), an aptamer has been identified for targeting TAR-DNA-Binding Protein 43 (TDP-43) [77]. 

Our laboratory has used a different strategy that targets other peptides that mimic critical pathological tau phosphorylation sites (Thr181, Ser 202, Thr231, Ser396/Ser404). This work identified an optimized DNA aptamer IT2a [81]. This epitope-specific DNA aptamer selectively recognizes Tau proteins with promising in vitro ability to inhibit Tau oligomerization and hyperphosphorylation. In a follow-up study, we delivered Tau aptamers into the animal brain by incorporating a second DNA aptamer (TfR1A) moiety that targets the TfR on the BBB. This leads to a circular construct, which reduces Tau overload and mitigates neurobehavioral dysfunction [110]. Exploring TfR to cross the BBB will be further discussed below.

In addition to Tau, Takahashi, and colleagues [85] and Song group [86] successfully selected RNA aptamers against the Aβ1-40 peptide, showing that the aptamers efficiently inhibit Aβ aggregation in vitro with therapeutic potential for treating AD. More recently, Lida et al. focused on preventing AD by destroying the formation of prion protein-Aβ oligomers [102]. They identified an anti-prion RNA aptamer, but more in vivo studies are needed to demonstrate its therapeutic ability. Interestingly, the Irie group from Kyoto University reported the first RNA aptamer targeting the toxic dimer of Aβ42 with significant prevention against the formation of Aβ42 aggregates and the related neurotoxicity [88] (Table 2).

Observation of oligomers and aggregates formed by disordered alpha-synuclein (α-syn) in neurons has been used to characterize Parkinson’s disease (PD). Similar to PD, the accumulation of aggregated α-synuclein protein in Lewy bodies and Lewy neurites results in dementia with Lewy bodies (DLB). Thus, reducing the aberrant aggregation of intracellular α-syn may prevent the progression of synucleinopathies such as PD and DLB [111]. The Zhang group has reported the identification of DNA aptamers targeting α-syn with high affinity [90]. Their aptamers present effective inhibitory ability on α-syn aggregation and promote its degradation in cell-based studies, rescuing cellular defects and showing potential for PD therapy. Other studies have shown different DNA aptamers that bind to α-syn fibrils and inhibit α-syn aggregation in in vitro models of PD and DLB [92,93,94]. Interestingly, Lobanova et al. selected DNA aptamer that binds to fibrillar aggregates of α-syn and Aβ detected in both serum and CSF in PD [95] (Table 2)

Exosomes have been employed to carry aptamers targeting α-syn [112] and deliver them into the PD animal model brain for improved investigation of in vivo effects. The aptamer-loaded exosomes were efficiently delivered into neurons in the animal model, strongly reducing in vivo neuropathological α-syn aggregates and alleviating behavioral deficits. Therefore, the anti-α-syn aptamers are promising therapeutic agents for the clinical treatment of synucleinopathies, such as PD. Further, exosomes have also been modified with aptamers targeting myelin, giving outstanding performance in promoting the remyelination process in mice models. This indicates the potential of preparing aptamer-based nano-medicines for managing sclerosis disease [113] (Table 2)

Both myelin basic protein (MBP) and autoantibodies against MBP have been targeted for aptamer development as potential multiple sclerosis (MS) therapy [114]. For glioma, aptamers have also been identified for CD20+B cells and the U87 glioma cell line/EGFRvIII. Neuro-aptamers target glutamate receptor GLuR1 [115], as well as the neuronal cell body and neurite-surface-located cell adhesion molecule (L1CAM) [101,116]. These neuro-aptamers may also be useful neuroscience research tools (Table 2)

Besides directly using aptamers as therapeutic agents, some researchers have linked the targeting ability of aptamers with neurotherapy in combination with other therapeutic agents, such as chemotherapy drugs. For example, a Gint4.T aptamer targeting platelet-derived growth factor receptor β has been widely used for glioblastoma neurotherapy. Monaco et al. anchored the aptamers to polymeric nanoparticles to cross the BBB, where they entered glioblastoma cells [98]. By loading with PI3K-mTOR inhibitor, a promising chemotherapeutic drug for treating glioblastoma, this aptamer-based nano-system demonstrated specific tumor accumulation and mTOR activity inhibition in a mouse model.

Similar studies were carried out by Shi et al. and Wang et al., who built tetrahedral DNA structures loaded with Gint4.T aptamers to facilitate BBB penetration and glioma engagement. These DNA structures were captured with either paclitaxel or doxorubicin to enable anti-glioma therapy [117,118] (Table 2).

## 5. In Vitro Neuro-Diagnostics Using Aptamers

Aptamers have been extensively used in vitro to detect various disease-specific biomarkers. Numerous authentic neuro-markers are found in nervous tissue, circulating in cerebrospinal fluid (CSF), and dissolved in the bloodstream of patients. These may be detected by appropriate probes for many purposes, such as the early diagnosis of neurologic conditions. Aptamer probes have many potential advantages here. Aptamers can be used for neuro-diagnosis by designing specific oligonucleotides to bind to disease-related biomarkers or pathogens using SELEX [119].

**Figure 4:**
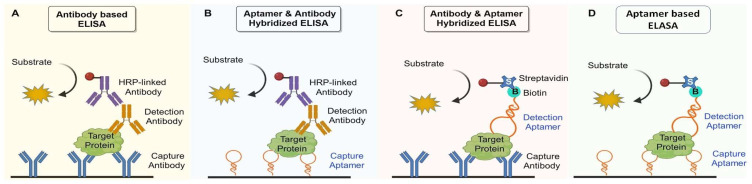
The setup of sandwich ELISA assay. (**A**) Classical antibody-based ELISA. The target protein is measured using two primary antibodies, a capture antibody and a detection antibody, followed by detection via HRP-linked antibody. (**B**) Antibody and aptamer hybridized ELISA. Capture antibody is replaced by capture aptamer. (**C**) Aptamer and antibody hybridized ELISA. The detection antibody is replaced by a biotinylated detection aptamer, which is recognized by HRP-streptavidin. (**D**) Aptamer-based ELASA. The target protein is measured using capture aptamer and biotinylated detection aptamer, followed by detection via HRP-streptavidin.

One traditional in vitro diagnostic technique uses aptamers in ELASA (enzyme-linked aptamer sorbent assay), a modified version of an ELISA [120]. Here, aptamers replace antibodies (Figure 4). One advantage of this technique is that the aptamer can be reused after heating and refolding [121]. Other ways to recycle the materials include washing with chaotropic reagents, surfactants, or chelating agents [122,123].

For example, Barbour et al. used the SOMA Scan Assay to analyze the CSF samples of 1128 multiple sclerosis patients using aptamer-based measurements. This proof-of-concept study proved that the analysis of biomarker ratios from CSF using aptamers is a promising method to differentiate RRMS from progressive MS. Twenty-one biomarker ratios distinguish RRMS from progressive MS with a validated area under the receiver operator characteristic curve of 0.91 (95% confidence interval, 0.80–1.00) [124]. Similarly, Farrar et al. established the β55 aptamer to detect the amyloid plaques in human AD brain tissue [125]. Another study with a sandwich assay format platform used aptamers to detect thrombin, which is involved in many diseases including Alzheimer’s [126]. These rely on two complementary thrombin-binding aptamers that bind at different sites on the protein, a 15-mer aptamer that has long been known, and a 29-mer aptamer [127,128].

## 6. In Vivo Imaging Using Neuro-Aptamers

Nuclear imaging, such as positron emission tomography (PET) and single-photon emission computed tomography (SPECT), uses molecular probes/ligands called “radiotracers” or “radiopharmaceuticals”. Once the probe is injected into the subject (human or animal), in vivo live imagers (SPECT or PET imager) detect the radiation produced by the probes. A visualized image of the probe localization is then produced, allowing both quantitative and qualitative assessment. PET is ten times more sensitive than SPECT, making it a powerful imaging technique [129]. On this front, PET visible radioisotopes have been used to label aptamers. The first successful in vivo aptamer diagnostic imaging trial targeted human neutrophil elastase, a marker of inflammation. It was labeled with 99mTc and injected intravenously in a rat model. The 99mTc-labeled aptamer imaged inflammation with a peak signal/noise ratio of 4.3 ± 0.6 at two hours, which was more significant than IgG with a peak signal/noise ratio of 3.1 ± 0.1 at three hours [130].

Magnetic resonance imaging (MRI) of disease targets can also be explored using MRI contrast agents such as gadolinium (Gd3+) which has strong paramagnetic properties. Thus, a target-specific aptamer can be derivatized with a Gd3+ chelating moiety, such as tetraazacyclododecane-1,4,7,10-tetraacetic acid (DOTA) [131]. Upon loading with Gd3+, the aptamer conjugate is visible on MRI. This method has been exploited in cancer imaging [132]. Last, a multi-fluorescein-labeled RNA 6 aptamer (β55) showed the localization of amyloid plaques in both the cerebral cortex and cerebral vasculature in the transgenic mouse model of AD. It was stable for at least 24 h. Taken together these studies show the feasibility of using aptamers as neuroimaging ligands [125].

## 7. Applying Experience from Non-CNS Therapeutic Aptamers towards Neuro-Aptamer Development

The potential for using aptamers in therapy was recognized in 1990 when Tuerk and Gold selected an RNA aptamer to target bacteriophage T4 DNA polymerase in the first SELEX experiment [2]. In the same year, Sullenger et al. reported the inhibitory effect of transactivation response (TAR)-containing sequences (TAR decoys) on HIV-1 viral infection in host cells. These TAR decoys (which perform as if they were aptamers) prevent the Tar protein from binding to the endogenous TAR RNA, inhibiting HIV gene expression and replication. TAR decoy RNA-mediated HIV inhibition was also suggested to be effective against natural HIV isolates despite their hypervariable nature because the replication of SIVmac was also inhibited in cells expressing HIV-1 TAR decoys [133]. These two independent discoveries suggested that specific nucleic acid sequences have potential for therapies in general.

Since then, aptamers have been studied in preclinical and clinical tests with different strategies employed for therapeutic applications, including aptamers as antagonists or agonists, and targeting ligands conjugated onto the drug carriers. In 2004, the first aptamer therapeutic, Macugen (Pegaptanib) was approved by the FDA, which is presently the only aptamer approved by the US FDA. It is effective as an anti-angiogenic medicine to treat neovascular (wet) age-related macular degeneration (AMD) [134].

Therapeutic targets for aptamers to date include thrombin [135] and nucleolin [136]. In addition, aptamers have been used to treat aging-related disorders [137], obesity and diabetes mellitus [138,139], cardiovascular diseases [140], infectious diseases [141,142], blood coagulation [143], bone diseases [144], immunological diseases [145] and cancers [146]. Experience from developing therapeutic aptamers for this range of targets, and knowledge regarding aptamer technology derived from this experience [147], will likely accelerate the development of aptamers toward managing neurological or neurodegenerative disorders.

## 8. Aptamers Targeting Transferrin Receptor 1 to Facilitate Drug Transport across the BBB

The BBB impedes the entry of blood-borne molecules and is necessary to maintain brain homeostasis. The BBB comprises endothelial cells joined by highly polarized tight junctions and supported by astrocytes and pericytes responsible for the isolation of the brain from peripheral circulation [148,149].

The highly restrictive nature of the BBB limits access to most bio-therapeutics, including antibodies, aptamers, and most (~98%) small-molecule drugs to the brain microenvironment [150]. Aptamers targeting CNS proteins may not cross the BBB unless they are combined with BBB-penetrating agents or unique loading structures, such as neuronal cell-derived exosomes, exploiting the TfR-based transcytosis, or some efficient nano-systems (such as tetrahedra or circular DNA structures and nanoparticles).

Structures implementing soft or solid nanomaterials improve the serum stability brain retention times of aptamers compared with the aptamers alone. This may mitigate the inherent drawbacks of rapid nuclease degradation and rapid renal clearance. This strategy may improve therapeutic efficacies [151].

Transferrin receptor 1, which is abundant in endothelial cells on the neurovascular material that forms the BBB, is considered a promising target for CNS delivery across the BBB. This has generated interest in developing antibodies [152] that bind TfR for CNS delivery [153,154]. Yu et al. reported the development of a low-affinity monoclonal antibody that binds TfR and therefore can cross the BBB to enhance the delivery of conjugated therapeutic antibodies that bind enzyme β-secretase (BACE1), a target for Alzheimer’s drugs.

Several TfR DNA and RNA aptamers are now known. Thus, Neufeld et al. first developed the GS24 DNA aptamer (50 nucleotides long [155]) for its ability to bind mouse transferrin receptor 1 (TfR1) as shown in Table 3. GS24 was subsequentially truncated and mutated by MacDonald et al. to give a short aptamer, TfRA1, only 14 nucleotides in length [156]. This team then further exploited a bispecific strategy that conjugated TfRA1 with the epithelial cell adhesion molecule (EpCAM) to treat brain cancer metastases [157].

Similarly, in our work, Teng et al. identified a DNA aptamer (IT2a) that binds tau protein and inhibits its phosphorylation [81]. We conjugated this aptamer to a mouse TfR aptamer, TfRA1, creating a cyclic bispecific aptamer. This circular construct binds both TfR and tau. It crossed the BBB and bound tau protein in both in vitro and in vivo models [110].

It is important to note that GS24 and TfRA1 are specific to mouse TfR and do not cross-react with their human counterparts. Maier et al. developed a TfR-specific RNA aptamer (WAZ), which targets the apical domain of the human transferrin receptor (hTfR) and does not interfere with transferrin binding. They propose that this is a critical feature for medical applications, as this aptamer will not interfere with the physiological functions of transferrin binding and uptake into the target cells (or through the BBB).

Last, Wu et al. reported a DNA aptamer (XQ-2d) selected against pancreatic ductal adenocarcinoma using the cell SELEX strategy [159]. The team subsequently found that its cellular target for XQ-2d was cell surface-bound CD 71, also known as TrfR1 [160].

## 9. Other Challenges in Aptamer-Based Therapeutic Development

Aptamers can potentially meet a critical unmet need for neurological disease therapeutics (Box 2). Because of their intrinsic non-immunogenicity and facile chemical synthesis, aptamers can be produced in higher purity and generally have longer shelf-time than antibodies. However, their inherent physicochemical characteristics, including short half-lives, susceptibility to nuclease degradation, rapid renal filtration, and impermeability to BBB, have limited the in vivo therapeutic potency of aptamers related to neurobiology [67,162,163].

Box 2 **Limitations of aptamers applied in neurodegenerative diseases and their potential solutions.**

Nuclease sensitivity
  - Chemical modification [164,165] (i) at the ends of the nucleic acid chain, (ii) of the nucleoside sugar ring, (iii) of the phosphodiester linkages.
  - Mirror-image aptamer (Spiegelmer [53]).
Renal excretion
  - Conjugation with a bulky moiety [166] to the end of aptamers, e.g., polyethylene glycol [167], cholesterol [168], protein [169], liposomes [170], or nanomaterials [171,172].
  - Multimerize single aptamers to give aptamer micelles [173], multivalent aptamers [174].
Binding affinity limited to nanomolar level
  - Increase the chemical complexity of aptamers [5].
Transfer across the BBB
  - Multivalent aptamer hybrid with TfR aptamer [110] (see above).
Therapeutic ability
  - Functionally guided SELEX (our group’s unpublished work).
Toxicity
  - More sophisticated mechanistic research is needed [175].

Moreover, the relatively low binding affinities of aptamers are still key issues in their clinical translation. Given that so far most therapeutic aptamers have acted as antagonists, an opportunity to chemically engineer antagonistic aptamers to improve biochemical and pharmacological properties to meet the criteria of human druggable therapeutics in neurodegenerative diseases is needed [67,176,177].

## 10. Critical Limitations and Potential Solutions

This section addresses some of the critical limitations of aptamers and potential solutions.

### 10.1. Selection Process

Aptamers are screened under in vitro conditions that often do not exactly replicate the conditions of clinical environments. This means that the structure, function, binding affinity, and specificity of an aptamer measured in vitro need not be the same in blood, tissue, or another complex clinical environment. Some methods, such as the Immunoprecipitation-Coupled SELEX (IP-Selex), have been developed to address this issue. In IP-Selex, the library and target are enriched under simulated physiological conditions. This process improves the properties of the selected aptamers under standard physiological conditions [45].

### 10.2. Nuclease Resistance and Renal Filtration

Aptamers are susceptible to nucleases that are prevalent in biological samples. The half-lives of unmodified aptamers are less than 10 min in vivo due to nuclease-mediated degradation. Chemically modified aptamers give improved in vivo resistance to plasma nucleases. The modifications are often introduced during DNA solid synthesis or PCR: (1) at the ends of the nucleic acid chain, (2) in the sugar rings of the nucleoside, (3) in the phosphodiester linkage, or (4) in the base of the nucleoside. Post-synthesis modifications also improve nuclease resistance, such as circularization, ligation, and conjugation.

Aptamers generally have a molecular mass range of 5–20 kDa. The molecular mass cut-off for the renal glomerulus is 30–50 kDa. Thus, aptamers are naturally susceptible to renal filtration, and are cleared from the circulatory system within ~5–10 min. Most chemical modification strategies for nuclease resistance do not slow the renal filtration of aptamers. Therefore, most principles for more efficient and cost-effective in vivo applications utilize aptamer-based nanoparticle platforms with synthetic or biological polymers or other bulky moieties [178].

### 10.3. Improvement in Binding Affinities

Most of the reported aptamers relevant to brain diseases bind their targets with Kds in the nanomolar range. It remains a challenge to obtain aptamers with higher affinity (in the pM range). In the typical building blocks of aptamers, nucleobases like ATCG/U provide only limited chemical diversity, especially when compared with the diversity of amino acids. As a result, the versatility of aptamers is highly constrained when targeting proteins and cells.

For example, it has been hypothesized that the lack of a nucleotide with a hydrophobic group that is found in many amino acids is a specific limitation to obtaining better binders. This hypothesis has led to the most effective modifications for a wide range of protein targets when selecting aptamers in the picomolar range.

Thus, scientists at SomaLogic added hydrophobic functional groups to the DNA bases to give modified aptamers, resulting in a dramatic increase in the affinity of the aptamer against a broad range of protein targets [73]. Hirao also developed a method for selecting DNA aptamers containing the four natural nucleotides and one unnatural nucleotide with the hydrophobic base 7-(2-thienyl) imidazo [4,5-b] pyridine (Ds). Hirao obtained DNA aptamers with affinities of 0.65 pM and 38 pM targeting two human proteins: vascular endothelial cell growth factor-165 (VEGF-165) and interferon-gamma (IFN-g), respectively [5]. The resulting aptamers are envisioned to be promising in targeting protein targets in animal models, given a more stable aptamer-protein complex.

### 10.4. Binding Does Not Always Equate to Therapeutic Functions

Most aptamers have been used in imaging or diagnosis, with only a few applied for therapeutic purposes [179]. Most SELEX experiments target affinity, and not downstream functionality. We can propose a shift in the selection paradigm to emphasize selecting aptamers based not only on binding to the targets, but also selected and enriched based on the ability of the aptamer to alter or affect the function of the biological targets (Functional SELEX) [180]. Such a strategy could provide a promising solution to speed up the generation of useful aptamers. Our team has recently developed the Functional SELEX-Based Selection of Tau Oligomerization-Inhibiting Aptamers with promising results [46].

## 11. Summary/Prospective

Since the introduction of SELEX in the 1990s, thousands of aptamers binding to different targets have been developed. Aptamers represent an interesting class of pharmaceuticals that lie between traditional organic pharmacophores and protein drugs in size, complexity, and synthetic accessibility. Aptamers can generally achieve the same affinities and specificities as therapeutic monoclonal antibodies, avoid the immunogenicity of protein drugs, and be developed more efficiently than is possible using high-throughput screening methods applied to small-molecule diversity.

However, traditional SELEX needs weeks to months to deliver the final aptamers. In addition, the success rate is low for obtaining aptamers with high affinity and, especially, high specificity, especially when highly “decorated” with hydrophobic groups in the aptamer.

New technology may reduce selection time and lead to better affinity and selectivity. Functionally guided SELEX may generate aptamers that have more direct medical uses. Though there are gaps in developing aptamers for clinical applications and risks associated with the pursuit of novel drug therapies, enormous potential remains for drugs that increase survival rates, reduce healthcare costs, enhance the quality of life, and support individualized therapies.

In the case of neurodegenerative and neurological diseases, the risk increases dramatically with age. More people are living longer, meaning that more people will be affected by neurodegenerative diseases in the coming decades. This situation adds to the urgency to improve our understanding of the causes of neurodegenerative diseases, and to develop new approaches for early diagnosis, prevention, and treatment. If the technology is further developed, aptamers offer a promising approach to managing and treating neurological and neurodegenerative diseases.

## Figures and Tables

**Table 1:** Advantages and limitations of aptamers and antibodies vs. small-molecule drugs.

Consideration	Small Molecule	Aptamer	Antibody
Structure	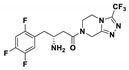	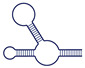	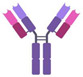
Size	<1 kDa	10~25 kDa	~150 kDa
Stability	Stable at R.T.	Stable at R.T.	Sensitive to temperature and pH changes
Cost	Low	Low	High
Immunogenicity	Low	Low	High
Ligand specificity	Medium (Kd nM to uM)	High (Kd pM to nM)	High (Kd pM to nM)
Toxicity	Mid-high toxicity	Not observed	Immune reaction
Administration	Oral, i.v.	i.v., s.c.	i.v., s.c.
Tissue penetration	Variable	Slow	Slow

**Table 2:** Known aptamers targeting neuroproteins or neuro-medicine-relevant proteins or cells.

Target	Aptamers (Name)	Dissociation Constant	Disease	Utility, Key Results	Ref.
GluR1 Ser845	A1, A2, A3 (RNA)	28–57 nM	Protein phosphorylation-related diseases	A2 binds GluR1 that inhibits GluR1/GluR1 containing AMPA receptor trafficking to the cell surface and abrogates forskolin-stimulated phosphorylation at GluR1 Ser845	[78]
(MAPK) Erk1/2	C5 (RNA)	10 nM	CNS disorders, Alzheimer’s disease, Stroke, Epilepsy	C5 selective to inhibit the mitogen-activated kinase pathway in neurons	[79]
β2-adrenoceptor (β2-AR) (GPCR)	A1, A2, A13, and A16 (RNA)	30.4–258.5 nM	-	RNA aptamers as allosteric GPCR modulators	[80]
Tau protein	IT(1–6), IT2a (DNA)	5.5–68 nM	Traumatic brain injury, Alzheimer’s disease	Aptamers inhibit tau phosphorylation and oligomerization	[81]
	Tau-1 (RNA)	~200 nM			[82]
	Aptamer 314 (DNA)	13 ± 3 nM		Aptamer binds Tau protein	[83]
β-Amyloid protein	β aptamers, e.g., β55 (RNA)	29–48 nM	Alzheimer’s disease	β55 aptamer binds amyloid plaques in AD brain tissue	[84]
	E1, E2, N1, N2, G2, etc. (RNA)	10.9–21.6 μM		N2 aptamer is used to build a luminescent aptamer-ruthenium complex system for the detection of Aβ	[85]
	Apt-GO (RNA)	0.1–10 μM		Apt-GO selectively detects Aβ1–42 in an AD SH-SY5 cell model	[86]
Aβ42	Aβ7-92-1H1	63.4 nM		Inhibits Aβ42 aggregation	[87]
Aβ42 dimer	E22P–AbD43 (RNA)	20 ± 6.0 nM		Aptamer inhibits the nucleation phase of the dimer and its associated neurotoxicity in SH-SY5Y human neuroblastoma cells.	[88]
Aβ40 oligomers	KM (RNA)	-		Aptamers bind with Aβ40 fibrils that may serve as amyloid recognition tools	[89]
α-synuclein protein	F5R1 (DNA)	2.40 nM	Parkinson’s disease	Blocks the aberrant cellular effects of the overexpressed α-synuclein in cells	[90]
	T-SO508 (DNA)	68 nM		T-SO508 can bind to soluble α-synuclein oligomers	[91]
	Apt11(DNA)	-	Parkinson’s disease and dementia with Lewy bodies	Apt11 aptamer binds to α-syn fibrils and inhibits α-syn aggregation in the in vitro model of PD and DLB.	[92]
	TMG-79 (DNA)	-		TMG-79 aptamer detects α-syn in Lewy body and PD-associated dementia.	[93]
	M5-15 (DNA)	14.3 nM		M5-15 aptamer detects α-syn in Lewy body and PD-associated dementia.	[94]
α-synuclein & amyloid-β (Aβ)	AD-PAINT (DNA)	500 nM–2 μM	Parkinson’s disease	AD-PAINT aptamer binds to fibrillar aggregates of α-syn and Aβ aggregates detected in both serum and CSF in PD	[95]
Dopamine	dopa2 (129 nucleotides); dopa2/c.1	2.8 µM 1.6 µM	Parkinson’s disease	Dopa2 and dopa2/c.1 are characterized to bind a dopamine affinity column; the dopamine binding site is obtained by secondary selection	[96]
Toll-like receptor 4 (TLR4)	ApTLR#1R, ApTLR#4F (DNA)	-	Stroke disease	Aptamers have a TLR4 antagonistic effect	[74]
	ApTOLL (DNA)	20 nM	Acute ischemic stroke	ApTOLL aptamer binds and antagonizes TLR4 and improves functional outcomes in AIS patients	[75]
Platelet-derived growth factor receptor β (PDGFRβ)	Gint4.T aptamer (RNA)	9.6 nM	Glioma	Aptamer binds to human DGFRβ ectodomain, causing a strong inhibition of ligand-dependent receptor activation	[97,98]
Myelin basic protein	MBPcl3 MBPcl9 (DNA)	-	Multiple Sclerosis	MBPc13 detects myelin-rich regions in paraffin-embedded mouse brain tissue; aptamer was found more sensitive than a commercial antibody. MBPcl3 blocks the binding of the antibody to MBP	[99]
Myelin basic protein (MBP) autoantibody	Apt2-9c (RNA)	1.2 ± 0.1 nM	Multiple Sclerosis	Apt2-9c provides proof-of-principle for the detection of MS-specific autoantibodies	[100]
L1-CAM (Neurites)	yly12 (DNA)	3.51 nM		Neurite-surface targeting and inhibitory effect on neurite outgrowth between cells	[101]
Prion protein (PrP)	R12 (RNA)	~10 nM	Creutzfeldt-Jakob disease; prion diseases	R12 binding to PrP results in the dissociation of PrP with Aβ.	[102]
	clone 4–9 (DNA)	113 nM		binds to PrP	[103]
	DP7 (RNA)	0.1–1.7 µM		Prion-protein-specific aptamer reduces PrPSc formation	[104]
	A1 (DNA)	232 nM		Aptamers modulate phase separation and promote PrP fibrillation	[105]
	R24 (RNA)	18 nM		R24 exhibited the lowest recorded IC50 and the highest anti-prion activity	[106]
Crossing BBB (target unknown)	A15 (RNA)	-	Neurological disorders or diseases.	In vivo SELEX (brain-penetrating aptamers)	[31]
CD20+B cells	TD05 (DNA)	256 nM	Glioma	TD05-488 can diagnose CNS lymphoma within 11 min of biopsy from xenograft brain tumor models	[107]
U87 glioma cell line/EGFRvIII	QD-A32 (DNA)	-	Glioma	QD-apt can penetrate the BBB and then selectively accumulate in the tumors through binding to EGFRvIII	[108]
The Regulator of calcineurin 1 (RCAN1)	R1SR13 (RNA)	0.3 µM	Down syndrome and Alzheimer’s disease	Inhibits the regulatory function of RCAN1 in NFAT and NF-kB signaling pathways	[109]
		0.23–30 nM	Acute ischemic stroke	R1SR13 aptamer alleviates the RCAN1.1 L-induced neuronal apoptosis in the human SHSY-5Y neuroblastoma cells and in the mouse model of AIS	[76]
TAR-DNA-Binding Protein 43 (TDP-43)	Apt-1 (DNA)	1 μM	Amyotrophic Lateral Sclerosis	Apt-1 aptamer binds to TDP-43 in the ALS model.	[77]

**Table 3:** Currently published transferrin receptor aptamers.

Transferrin Aptamer Name, Nucleotide Sequence	2-D Structure	Ref.
Mouse transferrin receptor-specific GS24, reduced to 50 nucleotides. Sequence (5′-3′): GCGTGTGCACACGGTCACAGTTAGTATCGCTACGTTCTTTGGTAGTCCGTTCGG	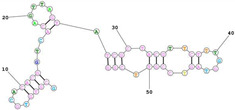	[155]
Target Mouse TfR Aptamer name: TfRA1 Truncated GS24; 14 nucleotides Sequence (5′-3′): GCGTGTGCACACGC	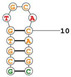	[156]
Human transferrin receptor specific C2. targets the apical domain of the human transferrin receptor (hTfR) Sequence (5′-3′): CAUCUCACAGAUCAAUCCAAGGCACCUCGUUAAAGGACGACUCCCUUACAUGCGAGAUG	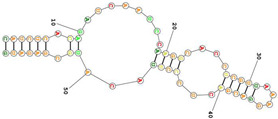	[158]
Aptamer name: Min2/Waz (RNA) non-competitive for Transferrin. Targets the apical domain of the human transferrin receptor (hTfR) Sequence (5′-3′): GGGUUCUACGAUAAACGGUUAAUGACCAGCUUAUGGCUGGCAGUUCCC	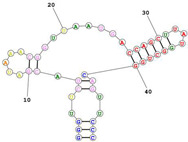	[141]
Human TfR (cell-SELEX) (XQ-2d Shares a Similar Binding Site on CD71 with Transferrin) Aptamer name: XQ-2d (DNA) Sequence (5′-3′): ACTCATAGGGTTAGGGGCTGCTGGCCAGATACTCAGATGGTAGGGTTACTATGAGC	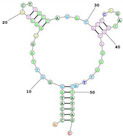	[159,160]
Human TfR (cell-SELEX) Aptamer name: HG1-9 (DNA) HG1-9 aptamer binds human TfR with affinity (Kd < 20 nM) and completes a same bind site of human TfR with transferrin. Sequence (5′-3′): GGATAGGGATTCTGTTGGTCGGCTGGTTGGTATCC	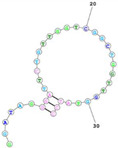	[161]
